# Three techniques for the determination of perindopril through derivatization with 4-chloro-7-nitrobenzo-2-oxa-1,3-diazole

**DOI:** 10.1186/s13065-023-00964-9

**Published:** 2023-06-22

**Authors:** H. Askar, Mohammed E.-S. Metwally, M. M. Tolba, Fatma A. Ali, M. E. Fathy

**Affiliations:** Pharmaceutical Analytical Chemistry Department, Faculty of Pharmacy, Mansoura University, Mansoura, 35516 Egypt

**Keywords:** Perindopril, NBD-Cl, Spectrophotometric, Spectrofluorimetric, HPLC, Dosage forms, Content uniformity testing, GAPI

## Abstract

In addition to its pure form, three accurate, rapid, and simple methods have been established for determining perindopril (PRD) in its tablet form. At pH 9.0 using a borate buffer, developing the three designated methods was successful according to the reaction between PRD and 4-chloro-7-nitrobenzo-2-oxa-1,3-diazole (NBD-Cl) and the formation of a chromogen (with a yellow color) measurable at 460 nm using the spectrophotometric method (Method I). In addition, the produced chromogen was assessed using the spectrofluorimetric method (Method II) at 535 nm following excitation at 461 nm. Afterward, the same reaction product was separated and determined using the HPLC method with fluorescence detection (Method III). A Promosil C_18_ stainless steel column (Q7 5 mm particle size, 250–4.6 mm) has proven suitable for separation. The mobile phase adjustment was made at pH 3.0, with a 1.0 mL min ^−1^ flow rate; its composition was methanol-sodium dihydrogen phosphate, 0.02 M (60: 40, v/v). Through concentration ranges of 5.0–60.0, 0.5–6.0, and 1.0–10.0 μg mL^−1^, the calibration curves were rectilinear for Methods I, II, and III, respectively, with limits of quantification (LOQ) of 1.08, 0.16 and 0.19 μg mL^−1^ as well as limits of detection (LOD) of 0.36, 0.05 and 0.06 μg mL^−1^. The developed methods were implemented to estimate PRD in tablets, and a comparison between the obtained outcomes utilizing the developed methods as well as obtained from the official method revealed that they were comparable. The official BP method was based on dissolving PRD in anhydrous acetic acid and titrating with 0.1 M perchloric acid, then the potentiometric determination of the end-point. The designated methods were also implemented in content uniformity testing with satisfying results. The reaction pathway proposal was speculated, and according to ICH Guidelines, the statistical evaluation of the data was performed. The three proposed methods were confirmed to be green, eco-friendly and safe to environment using Green Analytical procedure index (GAPI) method.

**Supplementary Information:**

The online version contains supplementary material available at 10.1186/s13065-023-00964-9.

## Introduction

**Fig. 1:**
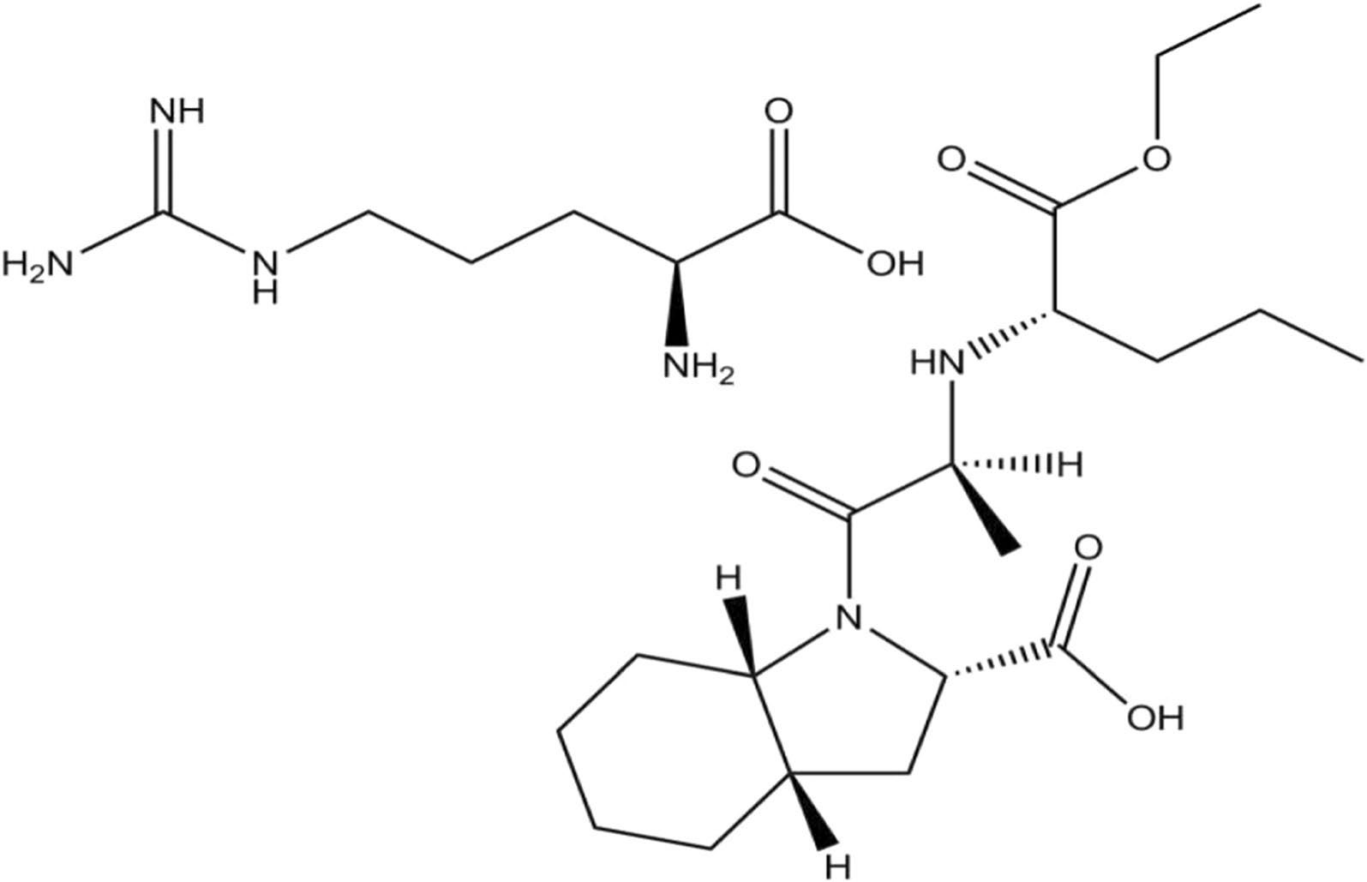
Structural formula of Perindopril arginine (PRD)

PRD, chemically known as (2S)-2-amino-5-carbamimidamidopentanoic acid; (2S,3aS,7aS)-1-[(2S)-2-{[(2S)-1-ethoxy-1-oxopentan-2-yl]amino}propanoyl]-octahydro-1H-indole-2-carboxylic acid (Fig. 1) [1]. PRD is used in heart failure and hypertension as it is an inhibitor of the angiotensin-converting enzyme (ACE). Its conversion to the active metabolite PRD in the body causes a low workload on the heart and a drop in blood pressure by the action of ACE inhibition. That leads to increased renin activity in plasma, decreased levels of angiotensin II in plasma, decreased aldosterone secretion, and decreased vasoconstriction [2].

PRD is officially represented in the British Pharmacopoeia (BP) [3]. Few methods for the estimation of PRD are reported in the literature, such as spectrophotometric [4–9], spectrofluorimetric [10–12], HPLC [4, 13–23], UHPLC [24], capillary electrophoresis [20, 23], gas chromatographic [25], and amperometric [26] methods.

**Fig. 2:**
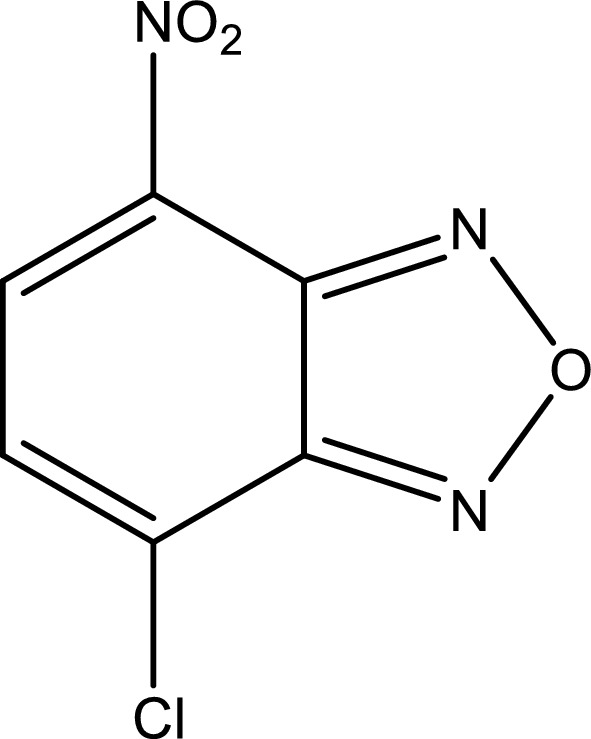
Structural formula of NBD-Cl

The electroactive halide reagent (NBD-Cl) (Fig. 2) is considered a target for nucleophiles in alkaline media. As reported by [27–35], many pharmaceutical compounds were determined utilizing NBD-Cl as an analytical chromogenic reagent.

This study aimed to develop precise, rapid, and simple spectrophotometric, spectrofluorimetric, as well as HPLC methods for PRD estimation in tablets in addition to their pure form. The validation of the developed methods via evaluating precision, linearity, limits of quantification, accuracy, as well as detection. In addition, the application of the developed methods to content uniformity testing was successful.

## Experimental

### Apparatus

- Establishing the spectrophotometric measurements (absorbance measurements) was done using the Shimadzu UV–Visible spectrophotometer (Kyoto, Japan; Model 1601PC), utilizing two matched cells (1 cm) made of quartz (recording range; 0–1.5).
- The spectrofluorimetric measurements were made utilizing an Eclipse fluorescence spectrophotometer from Agilent technologies as well as a flash lamp made of Xenon, adjusting the smoothing factor at 15. The wavelengths of excitation and emission were 461/535 nm. The voltage mode was high (800 v), and 5 nm was the slit width.
- A Shimadzu LC-20AD chromatograph from Japan was used to perform the HPLC separations. It was attached with a Rheodyne injector valve (20 μL loop) as well as an RF-10AXL fluorescence detector. The column used was Promosil C_18_ stainless steel column (Q7 5 mm particle size; 250–4.6 mm; Agela Technologies-USA). At room temperature, the operation of the column was done. The mobile stage components were methanol-sodium dihydrogen phosphate, 0.02 M (60: 40, v/v), with a 1.0 mL min^−1^ flow rate. Orthophosphoric acid was used for the adjustment of the pH of the mobile phase (pH 3.0). An HA membrane filter of 0.45 m (Millipore, Ireland) was used for the filtration of all chromatographic solutions. CBM-20A Communication Bus Module was utilized for connecting the instrument to the PC.
- Ultrasonic bath, SS 101 H 230 model-USA.
- To determine the pH of the used buffer solution, a digital pH meter (Consort NV P-90-Belgium) was utilized, and standard buffers were utilized for its calibration.

### Materials and reagents

All materials as well as reagents utilized were of analytical grade.

- Alamriya company (Alexandria, Egypt) supplied a pure sample of PRD arginine, with a purity of 99.6%.
- Coversyl® tablets labeled to contain 5 mg PRD arginine/tablet (Batch # 24462) or 10 mg PRD arginine/ tablet (Batch # 24316).
  They are products of Servier company (Lyon, France) and were obtained from a local pharmacy.
- Sigma-Aldrich (Louis, USA) was the company from which methanol (HPLC grade) was purchased.
- Sigma-Aldrich (Louis, USA) was the company from which NBD-Cl was obtained, and using methanol, a fresh stock solution of 0.2% NBD-Cl was prepared.
- From El-Nasr Chemical Co. (Cairo, Egypt), boric acid and sodium hydroxide were purchased. Both were mixed with appropriate volumes of 0.2 M aqueous solutions to obtain 0.2 M borate buffer. The used buffer solutions of pH 6.0–12.0 were prepared with the aid of a pH meter for adjustment of the required pH, and the buffer solutions were stable at room temperature.
- From El-Nasr Chemical Co. (Cairo, Egypt), conc. HCl (32%), sodium dihydrogen phosphate, and orthophosphoric acid were also purchased.

### Standard solutions

In Method I, the preparation of 400 µg mL^−1^ PRD standard solution was done using an ultrasonic bath, where the dissolution of 40.0 mg of PRD was done in distilled water (100.0 mL). In Methods II & III, the preparation of 200 µg mL^−1^ PRD standard solution was done using an ultrasonic bath, where 20.0 mg of PRD was dissolved in distilled water (100.0 mL). For obtaining the working solutions, the standard solutions were diluted as appropriate with distilled water (Methods I &II) or with the mobile phase (Method III). The standard solution stability following refrigeration was found to be at least two weeks.

## Procedures

### Calibration curve construction

#### Method I

Into a series of ten mL volumetric flasks, precisely calculated aliquots of PRD standard solution over a 5.0–60.0 µg mL^−1^ working concentration range were transferred. NBD-Cl (0.2%;1.0 mL) solution was added to all flasks before adding borate buffer (1.5 mL; pH 9.0), then mixed well. After that, heating the reaction solution was done (15 min at 50 ^o^C) in a thermostatically controlled water bath. They were subsequently cooled to ambient temperature before adding 0.2 mL of conc. Additionally, HCl was finally done. Using distilled water, the solutions were completed to the volume. An appropriate blank was prepared simultaneously. Then at 460 nm, the corrected reaction product absorbance was determined compared to the prepared blank solution. For calibration curve construction, the corrected absorbance (∆A) was plotted vs. final drug concentrations in µg mL^−1^. Alternatively, the equation of corresponding regression was obtained.

#### Method II

It was developed following the Method I procedure, except that 0.5–6.0 µg mL^−1^ was the final concentration range obtained following the standard solution’s dilution. At 535 nm after excitation at 461 nm, measurement of the relative fluorescence intensity (RFI) of the reaction product was done. For the calibration curve construction, plotting of RFI *vs.* the final drug concentrations (µg mL^−1^) was made. The corresponding regression equation was alternatively derived.

#### Method III

It was developed by also following the Method I procedure, except that 1.0–10.0 µg mL^−1^ was the final concentration range obtained after using the mobile phase to dilute the standard solution and to complete the volume of each flask to the mark. At ambient temperature (25 ^o^C), the separation was performed. After the injection of twenty microliters of aliquots (triplicate), plotting of the peak area (PA) was made *vs.* the final drug (µg mL^−1^) concentrations for the calibration curve construction. The corresponding regression equation was alternatively obtained.

### Analyzing the examined drug in commercial tablets

Weighing ten tablets was done, followed by pulverization. In a small conical flask, transferring the thoroughly mixed powdered tablets in a quantity of 40.0 mg of PRD was made using distilled water (90 mL) and extracted. In a 100 mL volumetric flask, the extract was filtered, then the conical flask was washed using distilled water in a few mLs. The washings were transferred to the 100 mL volumetric flask, and the solutions were made up to the volume using the same solvent. In a series of ten mL volumetric flasks, transferring of aliquots covering the working concentration range (Table 1) was made. Any further dilutions of the prepared solutions were made using either the mobile phase (Method III) or distilled water (Methods I & II). After that, *“Construction of the Calibration Curves”* was applied using any of the three developed methods. Tablets’ nominal content was estimated depending on the calibration curve that was previously plotted or the equation of corresponding regression.

**Table 1:** The developed methods’ analytical performance

Parameter	Method I	Method II	Method III
Concentration range (µg mL^−1^)	5.0–60.0	0.5–6.0	1.0–10.0
Intercept (a)	0.12	62.56	9.29
Slope (b)	0.02	139.30	77.95
Correlation coefficient (r)	0.9999	0.9999	0.9999
S_y/x_	2.3 × 10^–3^	2.93	1.72
S_a_	1.6 × 10^–3^	2.18	1.47
S_b_	1.0 × 10^–4^	0.64	0.22
%RSD	1.13	0.85	0.81
%Error	0.46	0.38	0.33
LOQ(µg mL^−1^)	1.08	0.16	0.19
LOD (µg mL^−1^)	0.36	0.05 0.06	

S_y/x_ = residuals’ SD

S_a_ = SD of the regression lines’ intercepts

S_b_ = SD of regression lines’ slopes

% Error = RSD%/√ n

LOQ = 10 S_a_/b

LOD = 3.3 S_a_/b

### Application to content uniformity testing

The exact procedure implemented for PRD determination in tablets was performed utilizing one tablet as a sample. After assaying ten various tablets, the Guidelines of the official USP [36] were applied to test their content uniformity.

## Results and discussion

**Fig. 3:**
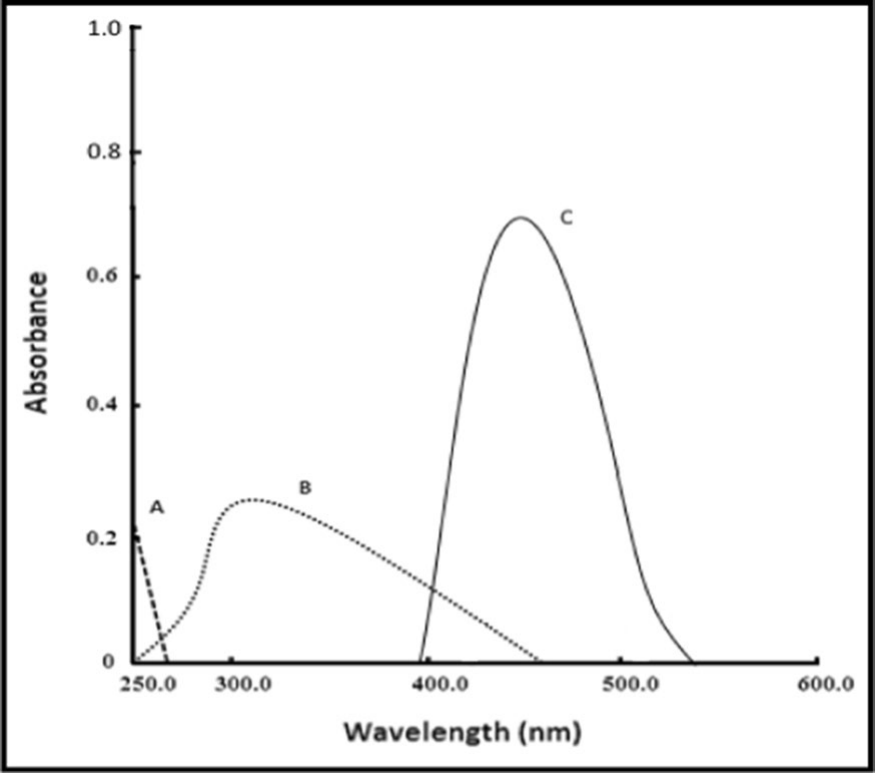
Absorption spectra of: **A** PRD only (40.0 μg mL^−1^). **B** blank NBD-Cl (0.2%; pH 9.0). **C** PRD reaction product(40.0 μg mL^−1^), with NBD-Cl (0.2%;pH 9.0)

**Fig. 4:**
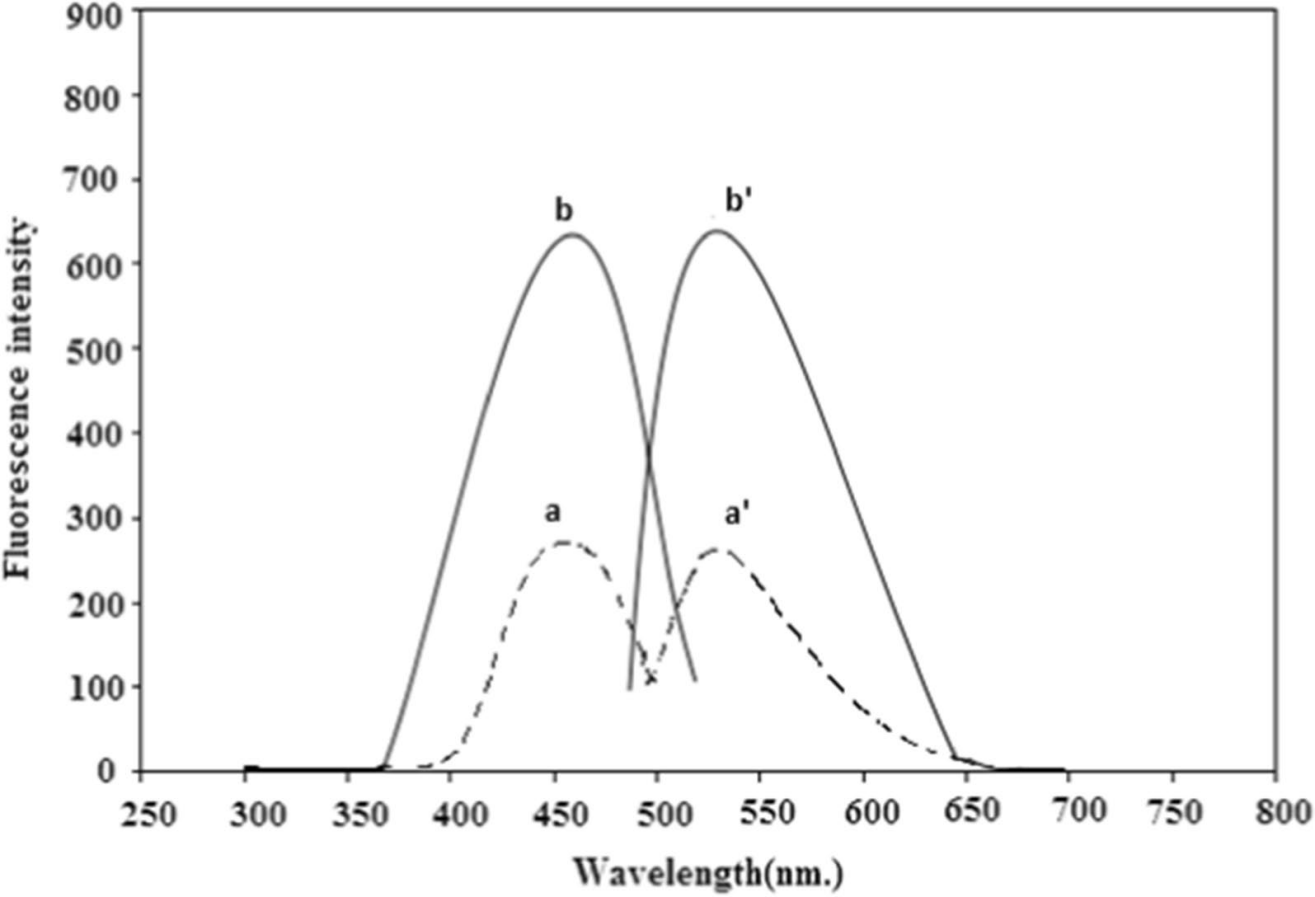
Excitation as well as emission spectra of: **a, a`** Blank NBD-Cl (0.2%;pH 9.0). **b, b`** PRD reaction product (4.0 μg mL^−1^), with NBD-Cl (0.2%;pH 9.0)

**Fig. 5:**
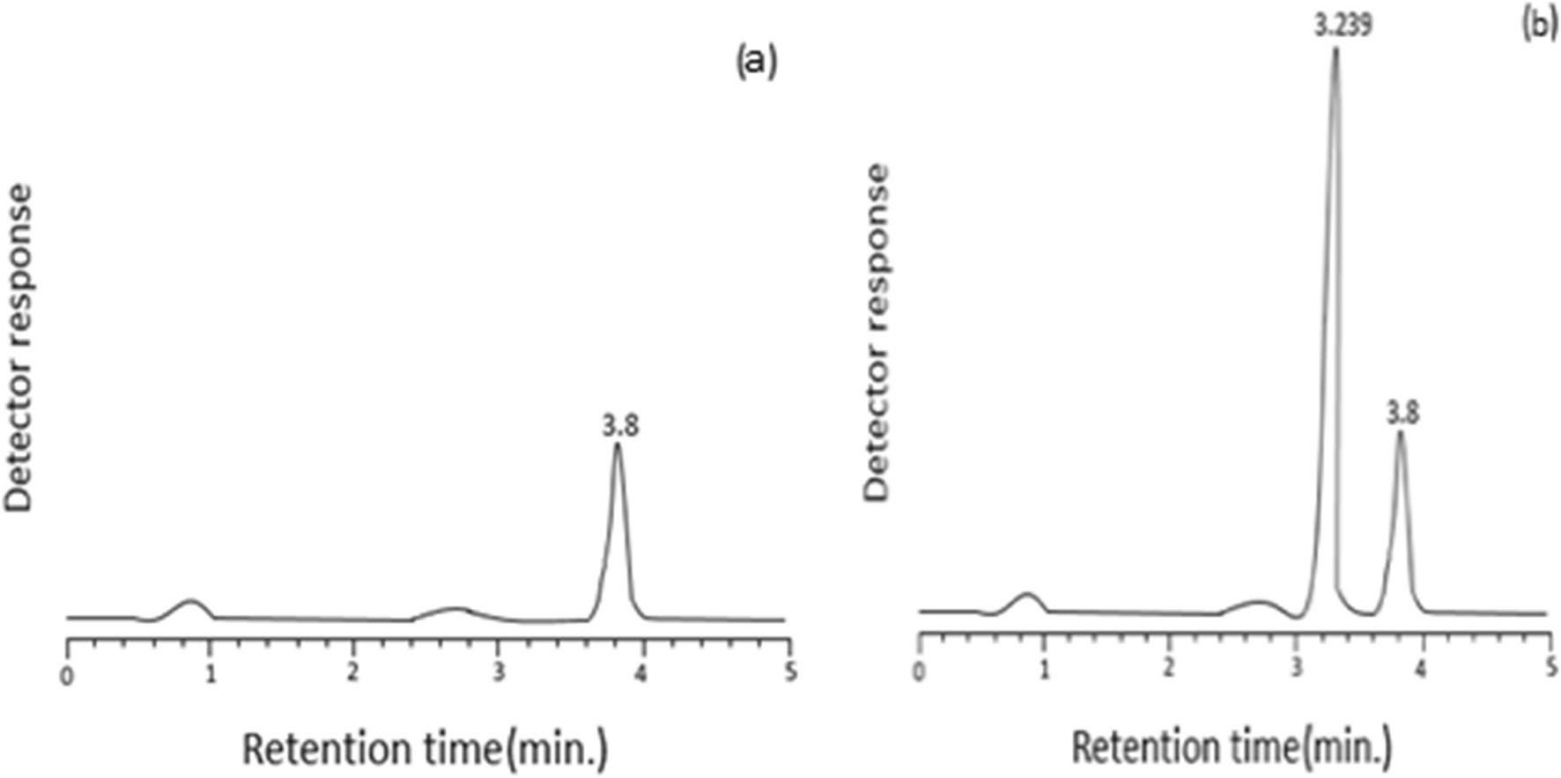
Typical chromatograms of **a** blank reagent; **b** derivatized drug (6.0 μg mL^−1^, 3.239 min;1 mL min.^−1^ flow rate)

PRD is a secondary amino compound, so it is a good nucleophile. For the determination of PRD, three simple, accurate, and selective methods were developed using a borate buffer (pH 9.0) depending on the reaction of nucleophilic substitutions between the studied drug and NBD-Cl, forming a chromogen of a yellow color. At 460 nm, measurement of the corrected absorbance (∆A) of the PRD reaction product with NBD-Cl (0.2%) was done (Method I) (Fig. 3). At 535 nm following excitation (at 461 nm), measurement of the RFI of the PRD reaction product with NBD-Cl (0.2%) was done (Method II) (Fig. 4). Then, developing HPLC method was done with fluorescence detection in order to estimate PRD (Method III) (Fig. 5).

The official BP method [3] was based on dissolving PRD in anhydrous acetic acid as well as titrating with 0.1 M perchloric acid, then the potentiometric determination of the end-point. The merits of the official method included simplicity, precision, and reproducibility, but it was not a sensitive method.

### Optimization of experimental parameters

Optimization of different experimental parameters was successfully done and resulted in the highest corrected absorbance (∆A) (Method I) or the RFI (Methods II &III) of PRD reaction product with NBD-Cl (0.2%). Chromatographic conditions were also optimized in Method III. An excellent separation was achieved for the blank reagent, and the derivatized drug peaks in a short retention time with the best resolution.

### Optimization for derivatization

The development, stability, and spectroscopic properties of the PRD reaction product of NBD-Cl (0.2%) were significantly affected by different experimental parameters optimized and studied carefully. These factors included 0.2% NBD-Cl volume, borate buffer volume, (0.2 M), pH of borate buffer (0.2 M), temperature, and reaction time, which were altered individually while keeping the remaining factors unchanged.

### Impact of NBD-Cl volume (0.2%)

**Fig. 6:**
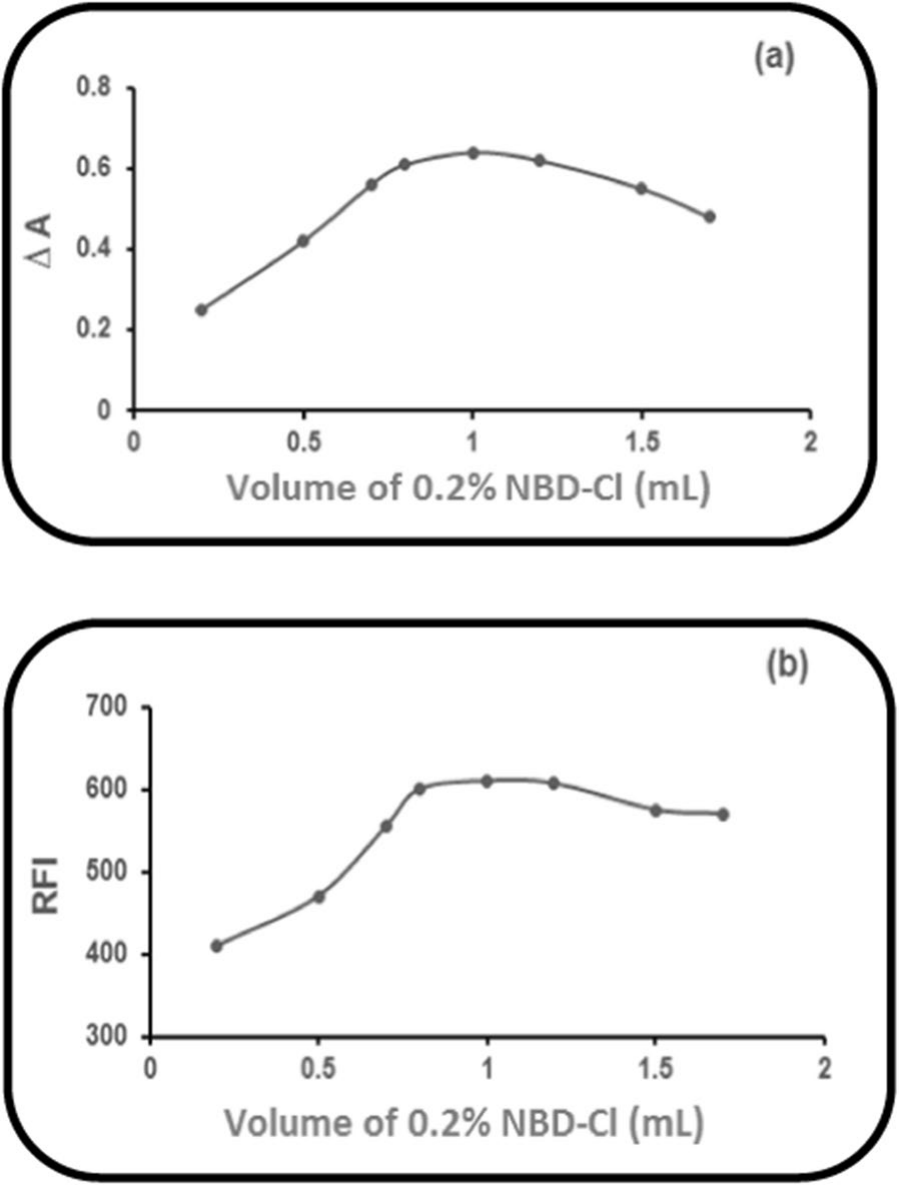
Impact of NBD-Cl volume (0.2%) on: **a** ∆A of PRD reaction product (60.0 µg mL^−1^), with NBD-Cl (0.2%). **b** RFI of PRD reaction product (6.0 µg mL^−1^), with NBD-Cl (0.2%)

Determination of the optimum volume of the reagent was necessary, so different NBD-Cl (0.2%) volumes were tried. It was found that increasing NBD-Cl (0.2%) volume caused an increase in the corrected absorbance (∆A) (Method I) or the RFI (Methods II &III) of PRD reaction product with NBD-Cl (0.2%) gradually until 0.8 mL and up to 1.2 mL, it remained constant. After that, a substantial decrease was observed (Fig. 6). Therefore, NBD-Cl (0.2%;1.0 mL) was selected throughout the study in the three proposed methods selected.

### pH impact of (0.2 M) borate buffer

**Fig. 7:**
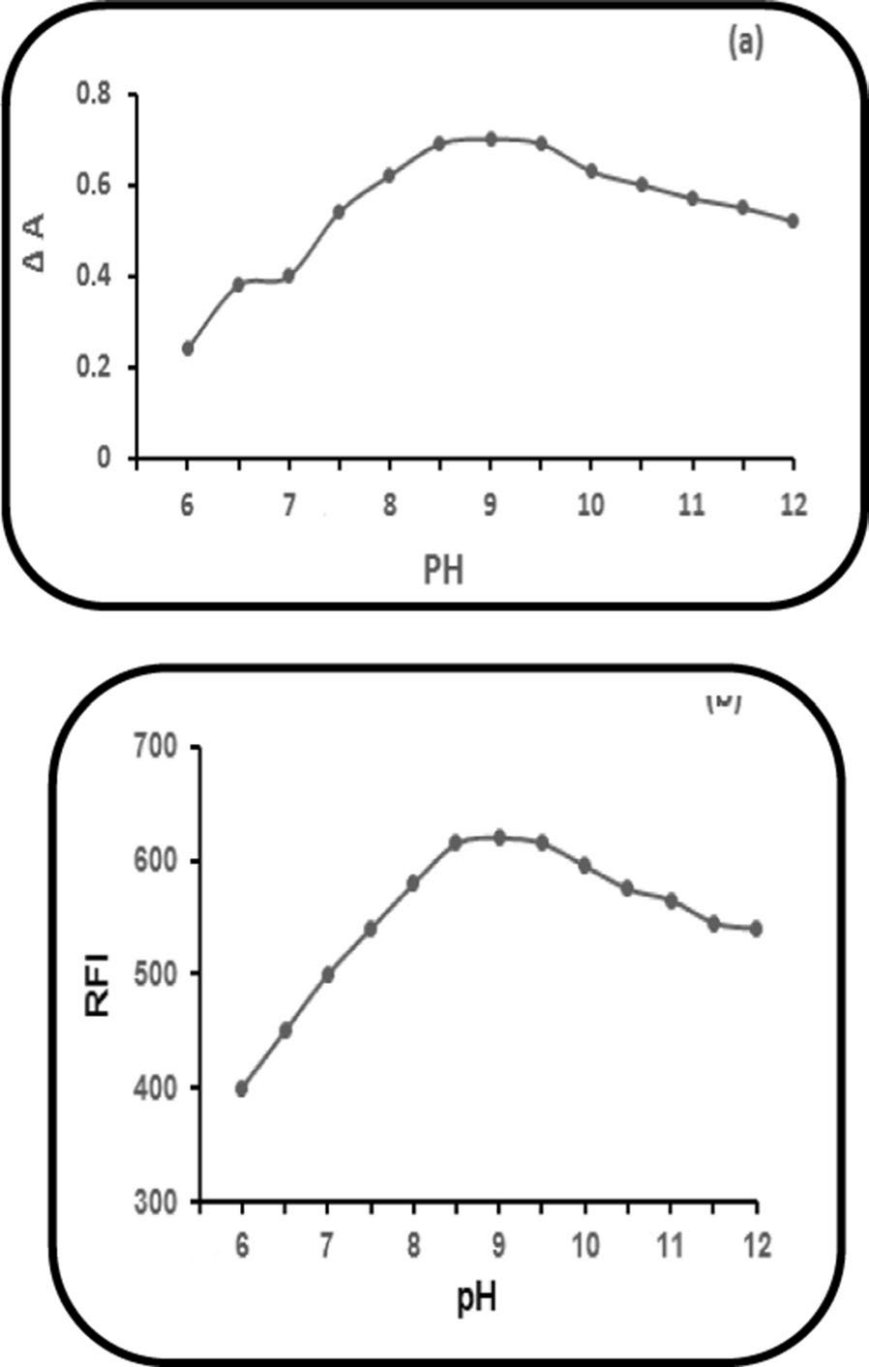
Impact of pH of 0.2 M borate buffer on: **a** ∆A of PRD reaction product (60.0 µg mL^1^), with NBD-Cl (0.2%). **b** The RFI of PRD reaction product (6.0 µg mL^−1^), with NBD-Cl (0.2%)

(6.0–12.0) was the pH range over which the influence of borate buffer’s pH (0.2 M) was studied. Elevating pH values resulted in elevated corrected absorbance (∆A) (Method I) or elevated RFI (Methods II &III) of the PRD reaction product with NBD-Cl (0.2%) until 8.8 and up to 9.2 and remained constant. After that, a noticeable decrease was observed (Fig. 7). Therefore, pH 9.0 of borate buffer (0.2 M) was used throughout the study in the three proposed methods as the optimum pH gave the highest values of ∆A and RFI.

### Impact of the volume of (0.2 M) borate buffer (pH 9.0)

**Fig. 8:**
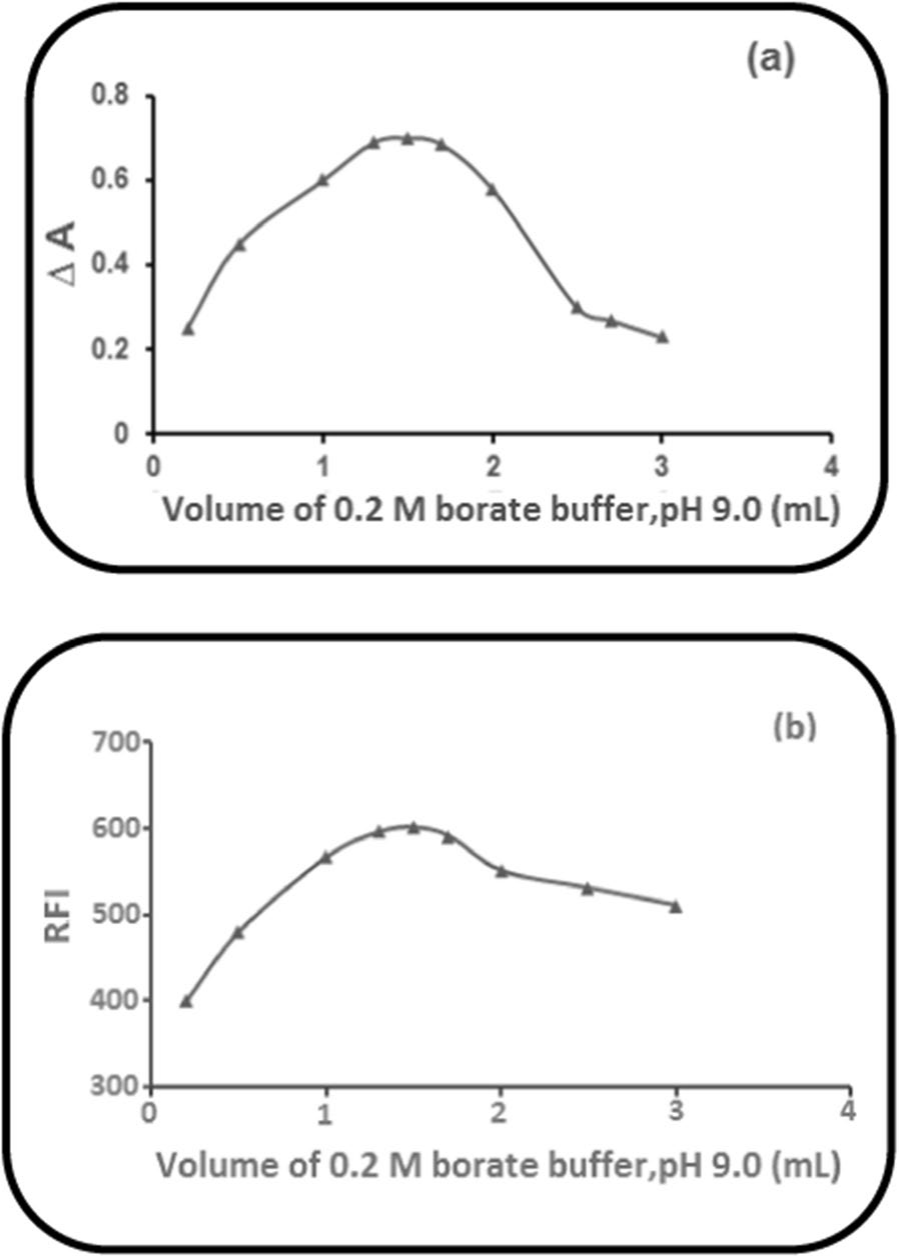
Impact of the volume of (0.2 M) borate buffer (pH 9.0) on: **a** ∆A of PRD reaction product of (60.0 µg mL^1^),with NBD-Cl (0.2%). **b** The RFI of PRD reaction product of (6.0 µg mL^−1^),with NBD-Cl (0.2%)

The optimum volume of the buffer was successfully determined. Increasing volume of 0.2 M borate buffer (pH 9.0) caused an increase in the corrected absorbance (∆A) (Method I) or the RFI (Methods II & III) of the reaction product of PRD with NBD-Cl (0.2%) gradually until 1.3 mL and up to 1.7 mL, it was found to remain constant. After that, a noticeable decrease was observed. Consequently, 1.5 mL of 0.2 M borate buffer (pH 9.0) was selected in the three proposed methods as it gave the highest values of ∆A and RFI (Fig.8).

### Temperature impact at heating time 15 min

**Fig. 9:**
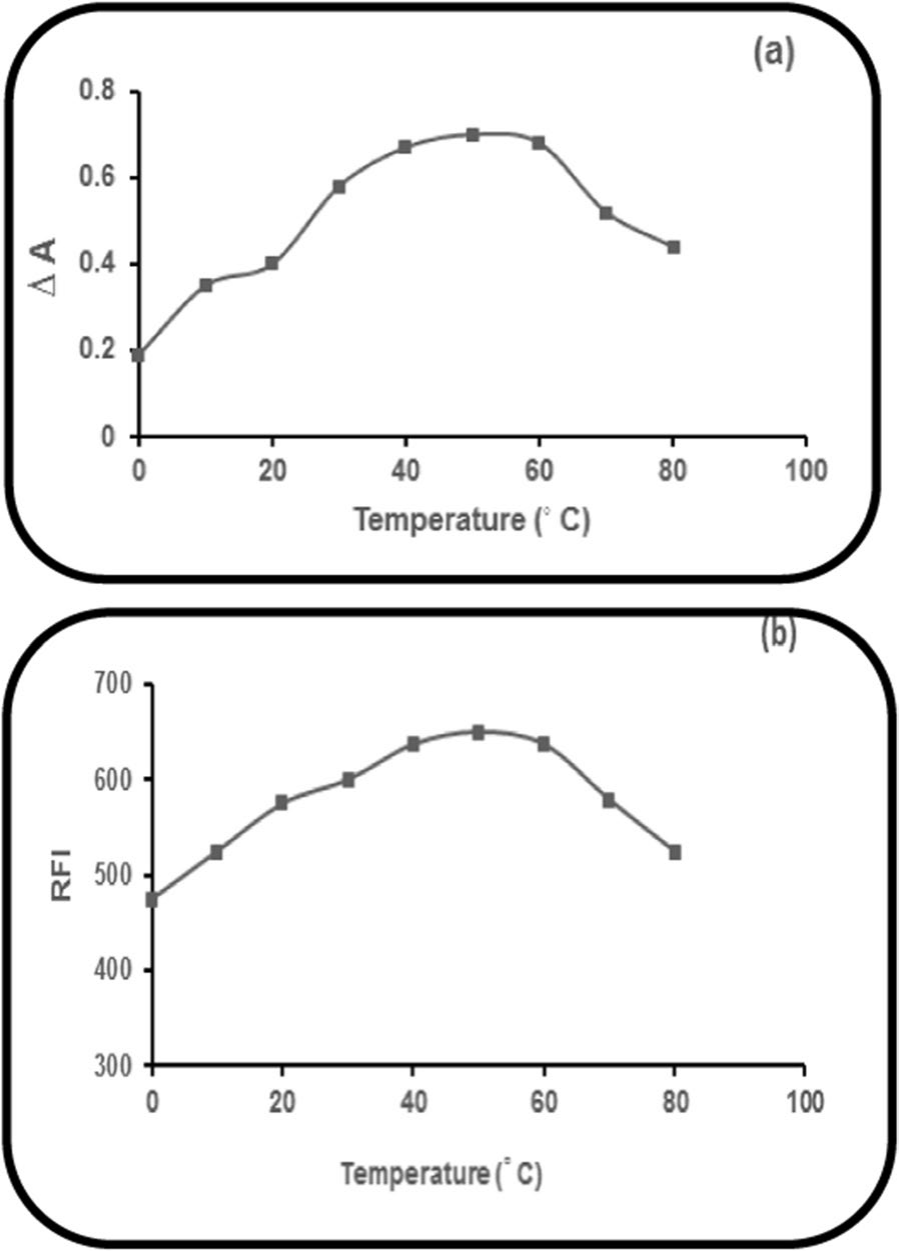
Temperature impact (at heating time 15 min.) on: **a** ∆A of PRD reaction product (60.0 µg mL^1^), with NBD-Cl (0.2%). **b** The RFI of PRD reaction product (6.0 µg mL^−1^), with NBD-Cl (0.2%)

Varying temperature settings were utilized upon an ongoing heating time (15 min). Elevating water bath temperature caused a subsequent increase in the corrected absorbance (∆A) (Method I) or the RFI (Methods II & III) of PRD reaction product, with NBD-Cl (0.2%) gradually until 48 ^o^C and up to 52 ^o^C remained constant. Afterward, a decrease in ∆A (Method I) or the RFI (Methods II&III) was noticed upon any further increase in the temperature. Consequently, it was found that the optimal temperature for the three proposed methods is 50 ± 2 °C (Fig. 9).

### Heating time impact at 50 °C

**Fig. 10:**
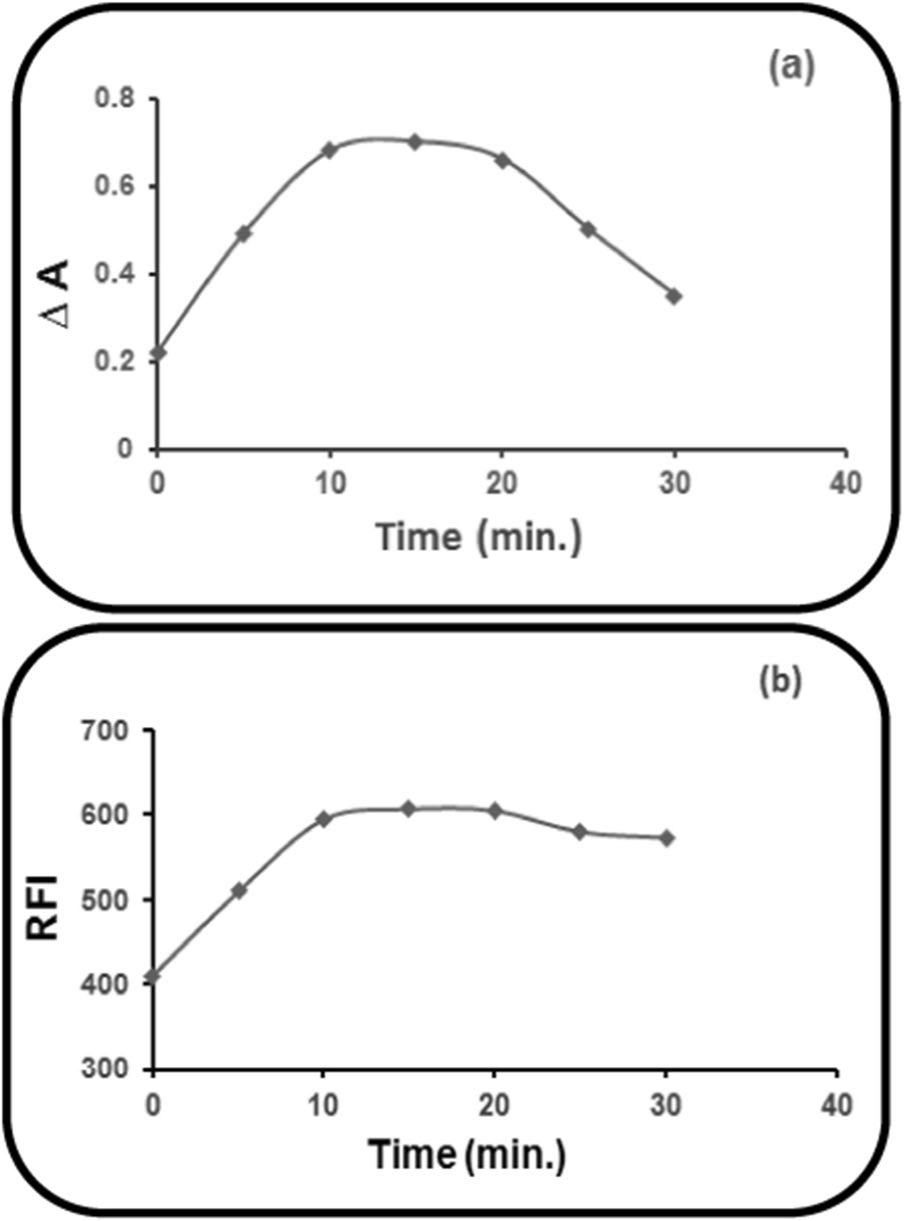
Heating time impact at 50 °C on: **a** ∆A of the PRD reaction product (60.0 µg mL^1^), with NBD-Cl (0.2%). **b** RFI of the PRD reaction product (6.0 µg mL^−1^), with NBD-Cl (0.2%)

Testing of different time intervals was done at 50 °C for determination of the optimum time of reaction. For the three developed methods, a heating time of 15 ± 2 min at 50 °C was found to give the highest corrected absorbance (∆A) (Method I) or the highest RFI (Methods II & III) of the reaction product of PRD with NBD-Cl (0.2%) and it was adequate for complete reaction (Fig. 10).

### Diluting solvent effect

It was necessary to use different solvents for dilution and to study their effect on the corrected absorbance (∆A) (Method I) or the RFI (Method II) of PRD reaction product with NBD-Cl (0.2%). Acetone, methanol, water, and acetonitrile were tried to be used as diluting solvents. In Methods I & II, the highest values of ∆A and RFI were retrieved utilizing distilled water. Therefore, the optimum diluting solvent in Methods I & II was finally distilled water. In Method III, the mobile phase was used for dilution.

### The reaction product stability

The formed PRD reaction product with NBD-Cl (0.2%) in the refrigerator was found to remain stable for 6 h at least.

### Optimization for separation

For Method III, peak separation and resolution of the blank reagent and the formed reaction product of PRD with NBD-Cl (0.2%) could be achieved using the HPLC method with fluorescence detection (at 535 nm) following excitation (at 461 nm). The mobile phase utilized was a blend of methanol-sodium dihydrogen phosphate, 0.02 M (60: 40, v/v).

Over the pH range of 2.0–7.0, the mobile phase pH was tested to study its effect on the blank reagent as well as the derivatized drug retention. Using the mobile phase of pH 3.0 resulted in the optimum separation.

Over the range of 20–80% (v/v), methanol concentration was also studied. Using the concentration 55–65% (v/v) resulted in the optimum resolution, then any further increase in methanol concentration produced asymmetric peaks (blank reagent and derivatized drug). Longer retention times were obtained at lower methanol concentrations.

The separation was possible in the case of using acetonitrile instead of methanol at the same percentage (60%, v/v), but the peaks were asymmetric. Hence, methanol was preferred.

### Chromatographic peak characterization

Depending on the described conditions, about 3.8 and 3.239 min were the times of retention for blank reagent and PRD after derivatization with NBD-Cl (0.2%), respectively. The peaks were separated with a resolution factor of about 2.75 in less than 5 min, a short retention time. A typical chromatogram of the blank reagent and the derivatized drug is shown in Fig. 5.

### Method validation

Based on ICH Q2(R_1_) guidelines [37], testing of intermediate precision, repeatability, accuracy, linearity, and specificity indicated the three proposed methods’ validity.

### Linearity and range

Regression plots indicated a linear relationship between values of ∆A, RFI, and PA, and concentrations of the drug over the ranges 5.0–60.0 µg mL^−1^, 0.5–6.0 µg mL^−1^, as well as 1.0–10.0 μg mL^−1^ for Methods I, II & III, respectively. For the construction of the three proposed methods’ calibration curves, ∆the A value in Method I or the RFI value in Method II and the PA value in Method III were plotted *vs.* drug concentrations (µg mL^−1^)_,_ according to the described conditions of the experiment. The data obtained from linear regression analysis for the three developed methods resulted in the subsequent three equations, respectively:

∆A = 0.120 + 0.014 C (r = 0.9999)

RFI = 62.55 + 139.30 C (r = 0.9999)

PA = 9.29 + 77.95 C (r = 0.9999)

Where ∆A is the corrected absorbance in a 1-cm cell, RFI = the relative fluorescence intensity, r is the correlation coefficient, C is the drug concentration (µg mL^−1^), as well as PA, is the peak area.

The correlation coefficients (r) of the regression equations were found to be of high values, indicated by the regression line statistical evaluation. Around the calibration curves, the points were of low scattering, and that was indicated by the standard deviations of the slopes (S_b_), of the intercepts (S_a_), and of the residuals (S_y/x_) that were of small values. In addition, the three designed methods were demonstrated to be highly precise as well as highly accurate, as proven by the percentage relative errors (% Error) and (%RSD) small values (Table 1).

### Limit of Detection (LOD) as well as Limit of Quantitation (LOQ)

Based on the ICH Q2 recommendation [37]_,_ LOQ values were estimated by detecting the lowest measured concentrations, and the calibration curves can not be linear below them. For the calculation of LOD values, the minimum detectable concentrations of analyte were established.

LOQ = 10 S_a_ / b LOD = 3.3 S_a_ / b.

Where S_a_ = SD of the calibration curves’ intercepts and b = slopes of the calibration curves.

LOQ & LOD values for Methods I, II, and III are depicted in Table 1.

### Accuracy

By comparing the outcomes of the proposed methods with the data attained from the official BP method [3], the three designed methods were found to be accurate.

Regarding accuracy and precision, respectively, no substantial difference was detected regarding the developed methods’ performance (Table 2). That was proved when the outcomes attained by the developed and the official methods for PRD were analyzed statistically utilizing the* F*-test and *t*-test [38].

**Table 2:** Assay outcomes for PRD determination in pure form by the official and proposed methods

Parameter	Method I			Method II			Method III			Official method [3] % Found
	The amount taken (µg mL^−1^)	Amount found (µg mL^−1^)	% Found	The amount taken (µg mL^−1^)	Amount found (µg mL^−1^)	% Found	The amount taken (µg mL^−1^)	Amount found (µg mL^−1^)	% Found	
	5.0	4.903	98.05	0.5	0.504	100.69	1.0	0.981	98.14	99.03
	10.0	9.871	98.71	1.0	0.997	99.70	3.0	3.024	100.8	98.74
	20.0	19.800	99.00	2.0	2.029	101.48	5.0	5.013	100.25	99.4
	40.0	39.420	98.55	4.0	4.045	101.12	7.0	6.975	99.64	
	60.0	59.466	99.11	6.0	6.060	101.00	10.0	10.016	100.16	
X`			98.68			100.80			99.85	99.06
± S.D			± 0.42			± 0.68			± 0.91	± 0.33
*t*			1.30			1.94			1.19	
*F*			1.60			4.17			9.39	

Each result is the average of three separate determinations

The tabulated values of *t* and *F* are (2.44) & (19.24), respectively, at p = 0.05 [38]

### Precision

The three proposed methods’ intermediate precision and reasonable repeatability were indicated by the SD of small values. For assessment of precision, three concentrations, as well as three replicates of each concentration, were determined in one day (Intra-day assay) and over three successive days (Inter-day assay) using the developed methods (Additional file 1: Table S1).

### Robustness of the methods

The methods developed were found to be robust by the constancy of the values of the corrected absorbance (Method I), the RFI (Method II), and the peak area (Method III), with the small alternations deliberated in the studied parameters during the experiment. For Methods I &II, there were small changes in 0.2 M borate buffer pH (9.0 ± 0.2), 0.2% NBD-Cl volume (1.0 ± 0.2 mL), 0.2 M borate buffer volume (1.5 ± 0.2 mL), temperature (50 ± 2^o^ C) and heating time (15 ± 2 min) (Additional file 1: Table S2a). For Method III, minor changes occurred in the mobile phase’s flow rate (1.0 ± 0.2 mL min^−1^), pH (3.0 ± 0.2), and conc. of methanol as an organic modifier (60 ± 2%, v/v) (Additional file 1: Table S2b). During experimental operations, these minor changes that may occur did not greatly affect the corrected absorbance, the RFI, or the peak area in Methods I, II, and III, respectively.

### Specificity

The common excipients of the tablet, including lactose monohydrate, magnesium stearate, and glycerol, showed no interference with the three methods developed. Consequently, the specificity of the methods developed was proved.

### Pharmaceutical applications

#### Tablet analysis

For PRD determination in tablets, the application of the developed methods was successful. In addition, *t*-test as well as *F*-test [38] were applied to compare the obtained oucomes statistically with the official BP method [3] (Additional file 1: Table S3).

#### Application to Content Uniformity Testing

The developed methods were found to be ideal for testing content uniformity. The British Pharmacopeia [3] and the United States Pharmacopeia [36] utilized the exact procedures for testing content uniformity.

As shown in Additional file 1: Table S4, excellent drug uniformity was demonstrated from the results. By comparing with the maximum allowed acceptance value (L1), acceptance values were found to be smaller than it.

#### Greenness Assessment of the proposed methods

The importance of greenness of analytical methods is human protection from chemicals having hazardous effects. In the green analytical method, there is no consumption of excessive energy, no production of harmful waste and toxic organic solvents can’t be used. Techniques for greenness assessment of analytical methods include Analytical eco-scale score, National Environmental Methods Index (NEMI) and the Green Analytical Procedure Index (GAPI) [40]. The three proposed methods were confirmed to be green, eco-friendly and safe to environment using Green Analytical procedure index (GAPI) method which is used recently [41].

A pictogram of GAPI method is applied for assessment of the effect of each stage of an analytical method on environment using colour scale of three levels: green, yellow and red, indicating low, medium and high impact on environment [41].

**Fig. 11:**
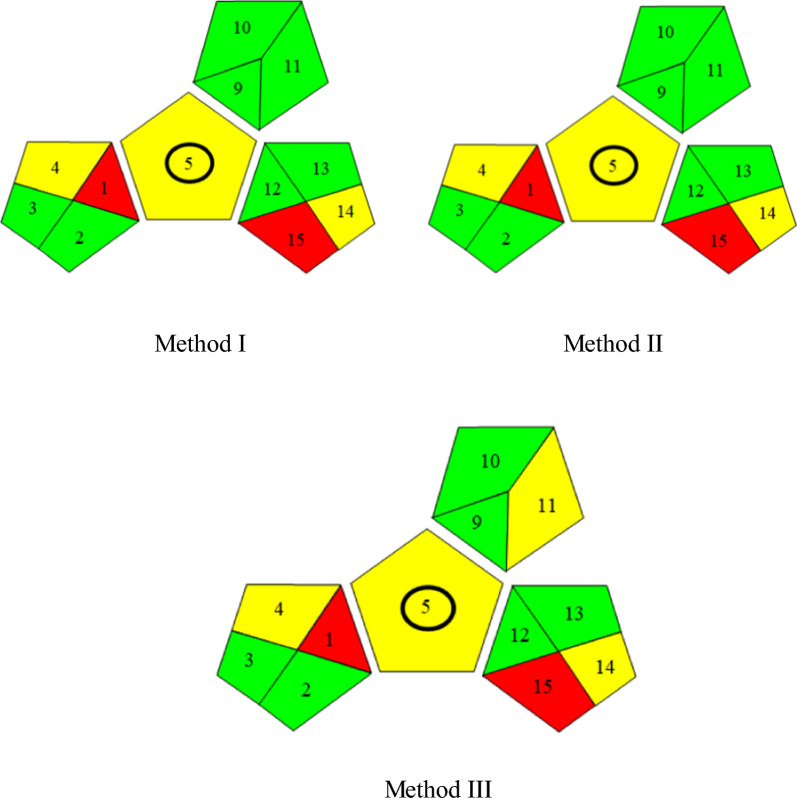
Greeness assessment results of the three proposed methods

For methods I and II, (Fig. 11) indicates fulfilling of GAPI major criteria except for fields 1,15 (red) because of the off-line sampling and no treatment of the waste, respectively. And fields 4,5,14 (yellow) which related to storage under normal conditions, carrying out the sampling procedure and the formation of 10 ml waste per sample, respectively. For method III, (Fig. 11) was illustrated as in methods I&II but field 11 was coloured yellow because of using methanol in mobile phase. GAPI was applied for PRD estimation in its tablets; the outcomes were coloured yellow, showing simple preparation (filtration) and the usage of green solvent (distilled water). The results proved that the proposed techniques were safe to the environment and humans. Also, they were green methods.

#### The reaction mechanism

**Fig. 12:**
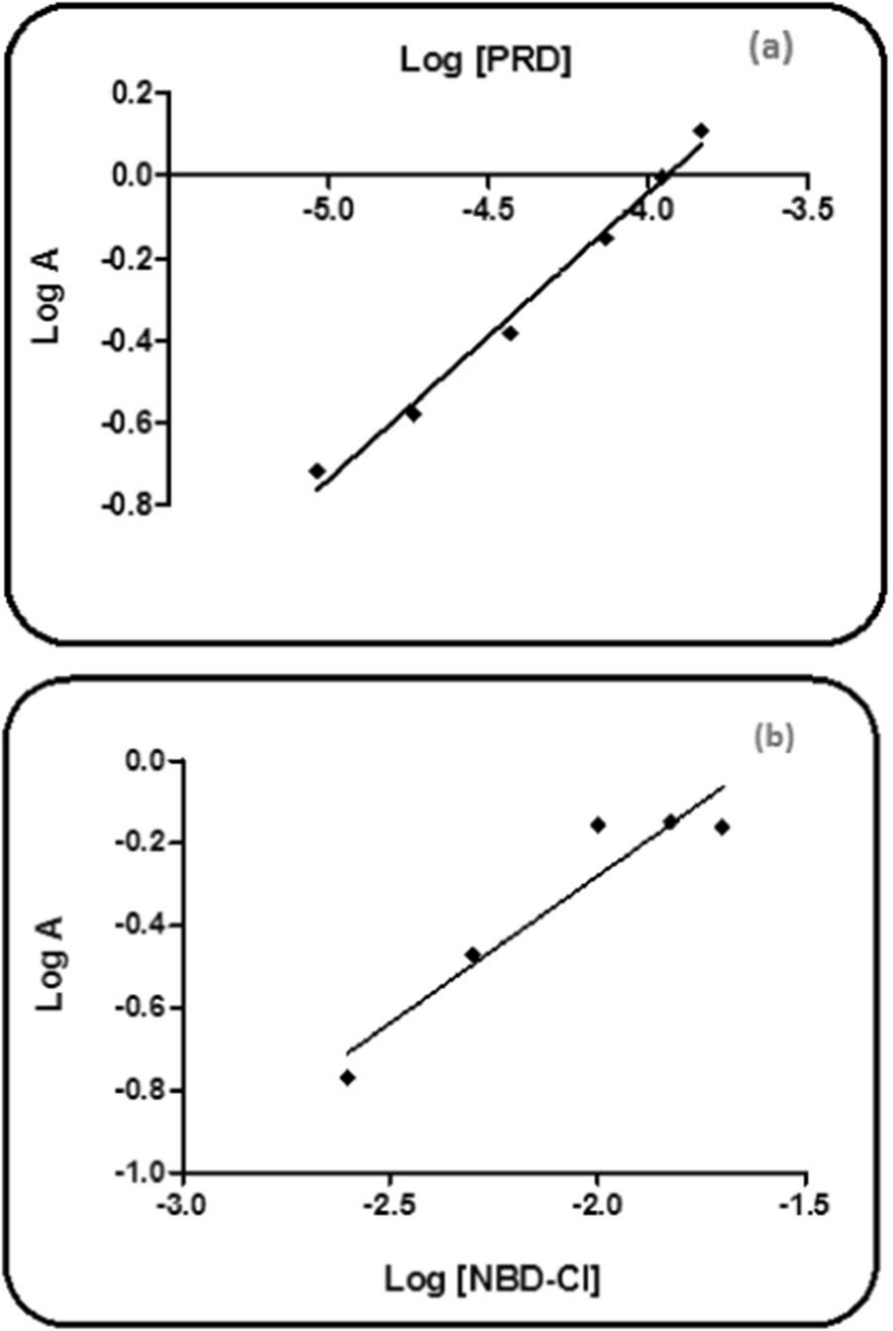
The reaction stoichiometry between PRD and NBD-Cl (0.2%) utilizing the limiting logarithmic method [33]. **a** Log [PRD] *vs* log A. **b** Log [NBD-Cl] *vs* log A

**Fig. 13:**
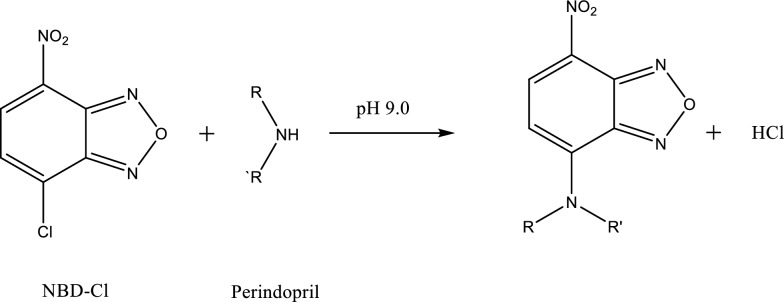
The proposed pathway for the reaction between PRD as well as NBD-Cl (0.2%)

Using the limiting logarithmic method [39], the reaction stoichiometry between the studied drug and NBD-Cl (0.2%) was examined. Two straight lines were obtained from the plots of log [PRD] vs. log A and log [NBD-Cl] vs. log A, and slope values were 0.694 and 0.714 for the two plots, respectively (Fig. 12). Therefore, the reaction’s molar reactivity is concluded to be 1:1 of the drug: NBD-Cl. A schematic proposal for the reaction pathway between PRD and NBD-Cl (0.2%) is shown in (Fig. 13) by analog to a previously published report [28] and depending on the molar ratio obtained.

## Conclusion

Three green, simple and accurate spectrophotometric, spectrofluorimetric, and HPLC methods were proposed for PRD determination in tablets in addition to their pure form. The common tablet excipients did not cause any interference. The three proposed methods were developed after using NBD-Cl (0.2%) for derivatization, then the reaction product was measured spectrophotometrically (Method I) and spectroflourimetrically (Method II). The same derivative was separated and then determined using HPLC with fluorescence detection (Method III). The application of the three developed methods to content uniformity testing was successful. The three proposed methods were simple and inexpensive from an economic point of view because they were developed with no need for extraction with an organic solvent. In addition, they have a greater linear range with high precision and accuracy. The methods developed can be utilized in quality control laboratories due to the excellent validation criteria. Our study’s limitation is that it included a reaction and required heating. In the future, we intend to apply the proposed methods to human plasma.

## Supplementary Information

**Additional file 1: Table S1.** Precision data for PRD by the three proposed methods. **Table S2a.** Robustness of Methods I and II using PRD and for the two methods, respectively. **Table S2b.** Robustness of Method III using PRD. **Table S3.** Assay results for the determination of PRD in its tablets by the proposed methods. **Table S4.** Results of content uniformity testing of PRD in tablets using the three proposed methods.

## Data Availability

All the data generated or analyzed during this study are included in this article.

## References

[1] O’Neil MJ, Smith A, Heckelman PE, Kinneary JF (2013). The Merk Index, an Encyclopedia of chemicals, drugs and biologicals.

[2] Sweetman SC (2014). Martindale: the complete Drug Reference.

[3] British Pharmacopoeia Commission. British Pharmacopoeia; TSO: London, Volume II; 2019.

[4] Karadurmus L, Gumustas M, Kurbanoglu S, Uslu B, Özkan SA (2018). A novel core-shell-based chromatographic method supported by ratio derivative spectrophotometry for the simultaneous determination of perindopril, indapamide, and amlodipine ternary mixtures. Turk J Chem.

[5] Rahman N, Rahman H, Haque SM (2017). Kinetic spectrophotometric method for the determination of perindopril erbumine in pure and commercial dosage forms. Arab J Chem.

[6] Masthannamma SK, Tejaswini IS, Saidulu P, Rambabu G (2015). Simultaneous equation method and absorption correction method for the estimation of perindopril erbumine and amlodipine besylate in bulk and in combined tablet dosage form using UV spectrophotometry. Int J Pharm Sci Res..

[7] Sakur AA, Balid B (2021). Direct spectrophotometric determination of perindopril erbumine and enalapril maleate in pure and pharmaceutical dosage forms using bromocresol green. Res J Pharm Tech.

[8] Unnisia A, Gopala Raju KV, Jyothi AN, Balaji K (2014). New spectrophotometric methods for estimation of perindopril erbumine in bulk and pharmaceutical formulations. J Sci Res.

[9] Rahman N, Rahman H (2011). Quantitative analysis of perindopril erbumine in pharmaceutical preparations by spectrophotometry via ternary complex formation with Zn (II) and eosin and charge transfer complexation with iodine. J Scpectrosc.

[10] Sakur AA, Chalati T, Fael H (2015). Selective spectrofluorimetric method for the determination of perindopril erbumine in bulk and tablets through derivatization with dansyl chloride. J Anal Sci Tech.

[11] Fael H, Sakur A (2015). Novel spectrofluorimetric method for the determination of perindopril erbumine based on fluorescence quenching of rhodamine B. J Fluoresc.

[12] Sakur AA, Chalati T, Fael H (2015). Selective spectrofluorimetric method for the determination of perindopril erbumine in bulk and tablets through derivatization with o-phthalaldehyde in presence of 3- mercaptopropionic acid. Int J Acad Sci Res..

[13] El-Bagary RI, Elkady EF, Mowaka S, Attallah MA (2017). A validated HPLC method for simultaneous determination of perindopril arginine, amlodipine, and indapamide: application in bulk and in different pharmaceutical dosage forms. J AOAC.

[14] Duraisamy K, Jaganathan KS, Krishna MV (2017). Method development and validation of HPLC tandem/mass spectrometry for quantification of perindopril arginine and amlodipine besylate combination in bulk and pharmaceutical formulations. Res Pharm Sci.

[15] Soujanya S (2017). Method development and validation of simultaneous estimation of perindopril and indapamide in tablet by RP-HPLC method. Indo Am J Pharm Res.

[16] Haque Sk M, Ratemi E (2016). Validated RP–HPLC method for the quantification of ACE inhibitor perindopril arginine. Eur J Pharm Med Res.

[17] Mastannamma SK, Tejaswini IS, Reehana SK, Saidulu P (2016). Stability indicating validated RP-HPLC method for simultaneous determination of perindopril erbumine and amlodipine besylate in bulk and pharmaceutical dosage form. Int J Pharm Sci Rev Res.

[18] Ega JK, Vadde R (2015). Validation and stability studies of developed HPLC method for estimation of amlodipine and perindopril in bulk and pharmaceutical dosage form. Int J Res App.

[19] Kiran BSS, Babu GR, Kumari MV, Kumar GV (2015). Stability indicating isocratic RP-HPLC method development and validation for indapamide and perindopril erbumine in pure and its combined tablet dosage form. Int J Pharm Sci Res..

[20] Valentin I, Silvia I, Gabriela CA, Lucia MD (2015). Analytical performance of an HPLC and CZE methods for the analysis and separation of perindopril erbumine and indapamide. Acta Medica Marisiensis.

[21] Dugga HHT, Peraman R, Nayakanti D (2014). Stability-indicating RP-HPLC method for the quantitative analysis of perindopril erbumine in tablet dosage form. J Chromatogr Sci.

[22] Yasmeen, Mamatha T, Gajula RG (2014). A novel RP-HPLC method development and validation of perindopril erbumine in bulk drug and tablet dosage form. Der Pharmacia Sinica..

[23] El-Gizawy SM, Abdelmageed OH, Derayea SM, Omar MA, Abdel-Megied AM (2014). Chiral separation of perindopril erbumine enantiomers using high performance liquid chromatography and capillary electrophoresis. Anal Methods.

[24] Al-Tannak NF (2018). UHPLC-UV method for simultaneous determination of perindopril arginine and indapamide hemihydrate in combined dosage form: a stability-indicating assay method. Sci Pharm.

[25] Lin SJ, Wu HL, Chen SH, Wen YH (1996). Derivatization-gas chromatographic determination of perindopril. Anal Lett.

[26] Van Staden JF, Stefan RI, Aboul-Enein HY (2000). Amperometric biosensor based on D-amino acid oxidase for the R-perindopril assay. Fresenius J Anal Chem.

[27] Belal F, Hosny MM, EL-Abassy OM, Elmansi H. (2021). Utility of NBD-Cl as an electrophilic reagent for the determination of the two antihypertensive drugs hydrochlorothiazide and minoxidil in dosage forms and human urine samples. Chem Pap.

[28] Walash M, El-Enany N, Askar H (2015). Validated spectrofluorimetric method for the determination of carbamazepine in pharmaceutical dosage forms after reaction with 4-chloro-7-nitrobenzo-2-oxa-1,3-diazole (NBD-Cl). J Biolog Chem Luminesc.

[29] Walash M, Belal F, El-Enany N, Elmansi H (2011). Development and validation of stability indicating method for determination of sertraline following ICH guidlines and its determination in pharmaceuticals and biological fluids. Chem Centr J.

[30] Martinc B, Roskar R, Grabnar I, Vovk T (2014). Simultaneous determination of gabapentin, pregabalin, vigabatrin, and topiramate in plasma by HPLC with fluorescence detection. J Chromatogr B.

[31] Martin MA, Lin B, Del Castillo B (1988). The use of fluorescent probes in pharmaceutical analysis. J Pharm Biomed Anal.

[32] Aboul-Enein Y, Elbashir A, Suliman F (2011). The application of 7-chloro-4-nitrobenzoxadiazole and 4-fluoro-7-nitro-2, 1, 3-benzoxadiazole for the analysis of amines and amino acids using High-Performance Liquid chromatography. Gazi Univ J Sci.

[33] Elbashir AA, Suliman FEO, Aboul-Enein HY (2011). The application of 7-chloro-4-nitrobenzoxadiazole (NBD-Cl) for the analysis of pharmaceutical-bearing amine group using spectrophotometry and spectrofluorimetry techniques. Appl Spectrosc Rev.

[34] Saleh HM, El-Henawee MM, Ragab GH, Abd El-Hay SS (2007). Utility of NBD-Cl for the spectrophotometric determination of some skeletal muscle relaxant and antihistaminic drugs. Spectrochim Acta Part A Mol Biomol Spectrosc.

[35] Ashour S, Khateeb M (2011). Kinetic spectrophotometric determination of pravastatin in drug formulations via derivatization with 4-chloro-7-nitrobenzo-2-oxa-1, 3-diazole (NBD-Cl). Arab J Chem.

[36] The United States Pharmacopeia 34^th^ and The National Formulary 29^th^, Rockville, MD, USA.

[37] ICH Harmonized Tripartite Guideline, Validation of Analytical Procedures, Text and Methodology, Q2(R1), Current Step 4 Version, Parent Guidelines on Methodology. 2005. http://www.bioforum.org.il/Uploads/Editor/karen/q2_r1_step4.pdf.

[38] Miller JN, Miller JC (2005). Statistics and chemometrics for analytical chemistry.

[39] Rose J (1964). Advanced physico-chemical experiments.

[40] Gamal M, Naguib IA, Panda DS, Abdallah FF (2021). Comparative study of four greenness assessment tools for selection of greenest analytical method for assay of hyoscine N-butyl bromide. Anal Methods.

[41] Płotka-Wasylka J (2018). A new tool for the evaluation of the analytical procedure: Green Analytical Procedure Index. Talanta.

