# Supplementary material for: Three techniques for the determination of perindopril through derivatization with 4-chloro-7-nitrobenzo-2-oxa-1,3-diazole
Source: BMC Chem. 2023 Jun 22;17(1):64. doi: 10.1186/s13065-023-00964-9 (PMC10288802; doi:10.1186/s13065-023-00964-9)
Supplement: Supplementary file 1 — Additional file 1: Table S1. Precision data for PRD by the three proposed methods. Table S2a. Robustness of Methods I and II using PRD and for the two methods, respectively. Table S2b. Robustness of Method III using PRD. Table S3. Assay results for the determination of PRD in its tablets by the proposed methods. Table S4. Results of content uniformity testing of PRD in tablets using the three proposed methods. [file 13065_2023_964_MOESM1_ESM.docx]

**Table S1: Precision data for PRD by the three proposed methods.**

| **Inter-day** | | | **Intra-day** | | | **Conc. (µg mL^-1^)** |  |
| --- | --- | --- | --- | --- | --- | --- | --- |
| **%Error** | **%RSD** | **X` ± S.D** | **%Error** | **%RSD** | **X` ± S.D** |  |  |
| 0.22 | 0.38 | 100.32 ± 0.38 | 0.23 | 0.40 | 101.41 ± 0.41 | 5.0 | **Method I** |
| 0.26 | 0.45 | 100.57 ± 0.46 | 0.20 | 0.34 | 99.92 ± 0.34 | 40.0 |  |
| 0.08 | 0.14 | 100.28 ± 0.13 | 0.16 | 0.28 | 99.28 ± 0.28 | 60.0 |  |
| 0.37 | 0.63 | 100.59 ± 0.64 | 0.35 | 0.61 | 100.53 ± 0.61 | 0.5 | **Method II** |
| 0.08 | 0.15 | 100.63 ± 0.14 | 0.25 | 0.44 | 99.05 ± 0.43 | 4.0 |  |
| 0.24 | 0.41 | 100.12 ± 0.42 | 0.07 | 0.11 | 100.87 ± 0.12 | 6.0 |  |
| 0.17 | 0.30 | 99.35 ± 0.30 | 0.16 | 0.27 | 100.27 ± 0.27 | 1.0 | **Method III** |
| 0.08 | 0.14 | 99.96 ± 0.14 | 0.10 | 0.17 | 100.60 ± 0.17 | 6.0 |  |
| 0.08 | 0.13 | 101.04 ± 0.13 | 0.11 | 0.18 | 99.16 ± 0.18 | 10.0 |  |

**N.B:** Each result is the average of three separate determinations.

**Table S2a: Robustness of Methods I and II using PRD (40.0 μg/mL) and (4.0 μg/mL) for the two methods, respectively.**

| **Parameter** | **Method I** | | | **Method II** | | |  |
| --- | --- | --- | --- | --- | --- | --- | --- |
|  | **Amount found**  **(µg mL^-1^)** | **%**  **Found** | **Amount found**  **(µg mL^-1^)** | | **%**  **Found** | | |
| **Reagent volume (mL)** |  |  |  | |  | | |
| 0.8 | 39.700 | 99.25 | | 3.958 | | 98.95 |  |
| 1.0 | 39.840 | 99.60 | | 3.965 | | 99.12 |  |
| 1.2 | 39.616 | 99.04 | | 3.942 | | 98.55 |  |
| X` |  | 99.30 | |  | | 98.87 |  |
| ± S.D |  | ± 0.28 | |  | | ± 0.29 |  |
| **pH of borate buffer** |  |  | |  | |  |  |
| 8.8 | 39.856 | 99.64 | | 4.018 | | 100.46 |  |
| 9 | 40.000 | 100.00 | | 4.007 | | 100.18 |  |
| 9.2 | 39.924 | 99.81 | | 3.997 | | 99.92 |  |
| X` |  | 99.82 | |  | | 100.19 |  |
| ± S.D |  | ± 0.18 | |  | | ± 0.27 |  |
| **Buffer volume (mL)** |  |  | |  | |  |  |
| 1.3 | 39.416 | 98.54 | | 4.049 | | 101.22 |  |
| 1.5 | 39.600 | 99.00 | | 4.026 | | 100.64 |  |
| 1.7 | 39.492 | 98.73 | | 4.057 | | 101.42 |  |
| X` |  | 98.76 | |  | | 101.09 |  |
| ± S.D |  | ± 0.23 | |  | | ± 0.41 |  |
| **Temperature (^o^ C)** |  |  | |  | |  |  |
| 48 | 39.950 | 99.88 | | 3.942 | | 98.54 |  |
| 50 | 40.100 | 100.25 | | 3.947 | | 98.67 |  |
| 52 | 40.290 | 100.73 | | 3.960 | | 99.00 |  |
| X` |  | 100.29 | |  | | 98.74 |  |
| ± S.D |  | ± 0.43 | |  | | ± 0.24 |  |
| **Time (min.)** |  |  | |  | |  |  |
| 13 | 39.456 | 98.64 | | 4.018 | | 100.45 |  |
| 15 | 39.720 | 99.30 | | 4.034 | | 100.85 |  |
| 17 | 39.928 | 99.82 | | 4.013 | | 100.32 |  |
| X` |  | 99.25 | |  | | 100.54 |  |
| ± S.D |  | ± 0.59 | |  | | ± 0.28 |  |

**N.B:** Each result is the average of three separate determinations.

**Table S2b: Robustness of Method III using PRD (6.0 μg/mL).**

| **Parameter** | **Amount found**  **(µg mL^-1^)** | **% Found** |
| --- | --- | --- |
| **pH of the mobile** **phase** |  |  |
| 2.8 | 5.994 | 99.90 |
| 3.0 | 5.976 | 99.60 |
| 3.2 | 5.997 | 99.95 |
| X` |  | 99.82 |
| ± S.D |  | ± 0.19 |
| **Conc. of organic modifier (methanol)**  **(% v/v)** |  |  |
| 58 | 5.949 | 99.15 |
| 60 | 5.968 | 99.47 |
| 62 | 5.952 | 99.20 |
| X` |  | 99.27 |
| ± S.D |  | ± 0.17 |
| **Flow rate (mL min^-1^)** |  |  |
| 0.8 | 6.032 | 100.54 |
| 1.0 | 6.018 | 100.30 |
| 1.2 | 6.018 | 100.30 |
| X` |  | 100.38 |
| ± S.D |  | ± 0.14 |
|  |  |  |

**Table S3: Assay results for the determination of PRD in its tablets by the proposed methods.**

| **Official method [3]** | **Method I Method II Method III** | | | | | | | | | | **Parameter** |
| --- | --- | --- | --- | --- | --- | --- | --- | --- | --- | --- | --- |
| **%**  **Found** | **% Found** | **Amount found**  **(µg mL^-1^)** |  | **Amount taken (µg mL^-1^)** | **%**  **Found** | **Amount found**  **(µg mL^-1^)** | **Amount taken (µg mL^-1^)** | **%**  **Found** | **Amount found**  **(µg mL^-1^)** | **Amount taken (µg mL^-1^)** |  |
| 99.48 | 100.00 | 1.000 |  | 1.0 | 99.20 | 0.496 | 0.5 | 99.80 | 4.990 | 5.0 | Coversyl^®^ tablets (5.0 mg PRD/ tablet) |
| 99.80 | 100.09 | 6.005 |  | 6.0 | 99.50 | 3.980 | 4.0 | 100.28 | 40.112 | 40.0 |  |
| 99.77 | 100.22 | 10.022 |  | 10.0 | 99.50 | 5.970 | 6.0 | 100.15 | 60.090 | 60.0 |  |
| 99.68 | 100.10 |  |  |  | 99.40 |  |  | 100.08 |  |  | X` |
| ± 0.18 | ± 0.11 |  |  |  | ± 0.17 |  |  | ± 0.25 |  |  | ± S.D |
|  | 2.13 |  |  |  | 1.98 |  |  | 2.24 |  |  | *t* |
|  | 2.55 |  |  |  | 1.04 |  |  | 1.97 |  |  | *F* |
|  | 99.57 | 0.996 |  | 1.0 | 101.20 | 0.506 | 0.5 | 99.85 | 4.993 | 5.0 | Coversyl^®^ tablets (10.0 mg PRD/ tablet) |
|  | 99.22 | 5.953 |  | 6.0 | 101.40 | 4.056 | 4.0 | 99.46 | 39.784 | 40.0 |  |
|  | 99.00 | 9.900 |  | 10.0 | 100.80 | 6.048 | 6.0 | 99.68 | 59.808 | 60.0 |  |
|  | 99.26 |  |  |  | 101.13 |  |  | 99.66 |  |  | X` |
|  | ± 0.29 |  |  |  | ± 0.31 |  |  | ± 0.2 |  |  | ± S.D |
|  | 2.15 |  |  |  | 2.13 |  |  | 0.13 |  |  | *t* |
|  | 2.64 |  |  |  | 2.99 |  |  | 1.22 |  |  | *F* |

**N.B.:**

All the obtained tablets are products of Servier Co (Lyon, France).

The tabulated values of *t* and *F* are (2.78) and (19.00) respectively, at p=0.05 [38].

Each result is the average of three separate determinations.

**Table S4:** **Results of content uniformity testing of PRD in tablets using the three proposed methods.**

| **Parameter** | **Percentage of the label claim** | |
| --- | --- | --- |
|  | **Coversyl^®^ 5 mg tablet** | |
|  | **Method I** | **Method II Method III** |
| **% Recovery** | 98.67 | 98.65 101.46 |
|  | 101.74 | 101.80 101.07 |
|  | 99.03 | 101.55 99.17 |
|  | 100.32 | 102.90 100.35 |
|  | 100.65 | 99.45 98.57 |
|  | 102.02 | 100.75 100.25 |
|  | 98.46 | 99.80 99.80 |
|  | 99.42 | 102.30 101.75 |
|  | 101.25 | 102.75 98.88 |
|  | 98.83 | 100.55 99.54 |
| **X`± SD** | 100.04 **±** 1.33 | 101.05 ± 1.45 100.08 **±** 1.09 |
| **% RSD** | 1.33 | 1.44 1.09 |
| **Acceptance value (AV)** | 3.19 | 3.48 2.62 |
| **Max. allowed AV (L1)[36]** | 1. 15.00 15.00 | |
